# Somatotropic Axis and Obesity: Is There Any Role for the Mediterranean Diet?

**DOI:** 10.3390/nu11092228

**Published:** 2019-09-16

**Authors:** Giovanna Muscogiuri, Luigi Barrea, Daniela Laudisio, Carolina Di Somma, Gabriella Pugliese, Ciro Salzano, Annamaria Colao, Silvia Savastano

**Affiliations:** 1 Dipartimento di Medicina Clinica e Chirurgia, Unit of Endocrinology, Federico II University Medical School of Naples, via Sergio Pansini 5, 80131 Naples, Italydani.lauidisio@libero.it (D.L.); robiniapugliese@gmail.com (G.P.); cirosalzano89@gmail.com (C.S.); colao@unina.it (A.C.); sisavast@unina.it (S.S.); 2 IRCCS SDN, via Gianturco 113, 80143 Naples, Italy; cdisomma@unina.it

**Keywords:** obesity, GH deficiency, IGF-1, insulin resistance, Mediterranean diet

## Abstract

Obesity is associated with reduced spontaneous and stimulated growth hormone (GH) secretion and basal insulin-like growth factor I (IGF-1) levels—which in turn is associated with increased prevalence of cardiovascular risk factors. The aim of this study was to investigate: (1) the association of somatotropic axis with cardiometabolic status; (2) the association of somatotropic axis with the Mediterranean diet and nutritional pattern in people with obesity. Cross-sectional observational study was carried out in 200 adult women, aged 36.98 ± 11.10 years with severe obesity (body mass index—BMI of 45.19 ± 6.30 kg/m^2^). The adherence to the Mediterranean diet and the total calorie intake was assessed. Anthropometric measurements, body composition and biochemical profile were determined along with Growth Hormone (GH)/Insulin like Growth Factor 1 (IGF-1) axis and insulin resistance (homeostatic model assessment for insulin resistance—HoMA-IR). The enrolled subjects were compared after being divided according to GH peak response and according to IGF-1 standard deviation scores (SDS). Derangements of GH peak were detected in 61.5% of studied patients while IGF-1 deficiency was detected in 71% of the population. Both blunted GH peak response and IGF-1 SDS were indicators of derangements of somatotropic axis and were associated with comparable results in terms of cardiometabolic sequelae. Both GH peak and IGF-1 levels were inversely associated with anthropometric and metabolic parameters. The adherence to the Mediterranean diet predicts GH peak response. Fatty liver index (FLI), fat mass (FM) and phase angle (PhA) were predictive factors of GH peak response as well. In conclusion derangements of somatotropic axis is associated with a worse cardiometabolic profile in people with obesity. A high adherence to the Mediterranean diet—and in particular protein intake—was associated with a better GH status.

## 1. Introduction

The GH/IGF-1 axis is finely regulated at multiple steps by neuroendocrine mediators, tissue and soluble receptors and carrier proteins. Growth hormone (GH) secretion—either spontaneous or evoked by provocative stimuli—is markedly disrupted in obesity [1,2]. The alterations of GH/IGF-1 axis in obesity are characterized by the decrease in the half-life of GH along with a reduction in both frequency and amplitude of GH secretory bursts. These phenomena are associated with an increased GH metabolic clearance rate which at the end results in low plasma GH levels [3]. A worse cardiovascular risk profile and body composition with increased cardiometabolic consequences has been detected in obese individuals with a low GH status compared to obese individuals without impairments in the somatotropic axis [1,4,5,6,7]. Moreover, the alterations of GH/IGF-1 axis could encourage the onset of sarcopenic obesity which in turn could represent a further obstacle for physical activity, thus worsening cardiovascular risk and mineral metabolism [8,9]. The low GH status in obesity is considered to be an acquired functional defect; in fact, it has been demonstrated to be reversed after weight loss process. The low GH status seems to be often associated with visceral obesity leading us to hypothesize that fat deposition at abdominal site could be one of the most determining player of GH secretion derangements in obesity. FFA (free fatty acids), which are known to be tightly related to visceral adipose tissue, have been reported to have a role in lowering GH secretion, and the administration of antilipolytic agent (acipimox) restored a normal GH secretion through the inhibition of FFA [10]. Further, dysregulation of GHRH (GH releasing hormone), somatostatin, ghrelin pathways, and hyperinsulinemia contribute to blunt GH secretion in obesity [11]. However, GH/IGF-1 axis function could be regulated by nutritional components as well [12]. Several studies have consistently reported that a high milk intake exerts relevant effects on somatotropic axis by increasing both growth hormone (GH) and insulin-like growth factor-1 (IGF-1) secretion [13,14,15]. Moreover, it has been reported that short-time breastfeeding has been associated with an increased risk of developing childhood obesity [16]. Currently, no studies on the regulation of the somatotropic axis by dietary factors, mainly a Mediterranean diet, were carried out in people with obesity. The aims of this study are two-fold: firstly, to evaluate the association between cardio metabolic status and somatotropic axis in obese individuals; secondly, to investigate the association between the Mediterranean diet and GH secretory status in these subjects.

## 2. Materials and Methods 

### 2.1. Design and Setting

This is a cross-sectional observational study carried out at the Department of Clinical Medicine and Surgery of the University of Naples Federico II (Italy). The procedures used were in accordance with the guidelines of the Helsinki Declaration on human experimentation. The study was approved by the Ethics Committee of the Federico II University Medical School of Naples (n 5/14). The aim of the study was clearly explained to all the enrolled subjects.

### 2.2. Population Study

Two hundred adult female individuals (>18 years of age) were enrolled after providing written informed consent. None of the study participants had any known relevant endocrine or non-endocrine diseases. Eligible participants for the study were adult subjects aged 18–75 years with a BMI (body mass index) >35 kg/m^2^. The participants had normal liver, cardiopulmonary and kidney functions as determined by medical history, physical examination, electrocardiogram, urinalysis blood tests for blood urea nitrogen, creatinine, uric acid, albumin, aspartate aminotransferase, and alanine aminotransferase. Other exclusion criteria were alcohol or drug abuse and history of allergy or intolerance to olive oil. Subjects following a specific dietary regimen for any reason were excluded from the study as well. The study was conducted without support from the pharmaceutical industry.

### 2.3. Dietary Assessment

A seven day food record was used in order to perform dietary assessment [17,18]. The instructions on how to fill the diary was provided by nutritionists, whom recommended participants to report the previous day’s intake. Participants prospectively filled the diary regarding the rest of the week. The enrolled patients brought back the diary records to the nutritionist who made further enquiries if needed. A commercial software was used to store data [19]. Based on quantities and qualities of food intake, the software calculated not only the daily caloric intake but the amount of macronutrients as well (protein; total, complex, and simple carbohydrates; fibers; total fat, saturated fatty acids (SFA), monounsaturated fatty acids (MUFA), polyunsaturated fatty acids (PUFA): n-6 PUFA, n-3 PUFA and n-6/n-3 PUFAs ratio; and cholesterol). As previously reported [20,21,22,23,24,25], the adherence to the Mediterranean Diet was assessed by a previously validated 14-items questionnaire) PREDIMED (PREvention with MEDiterranean Diet) questionnaire [26]. The questionnaire was performed by a qualified nutritionist through a face-to-face interview to all the enrolled subjects. Scores of 1 and 0 were assigned for each item. The PREDIMED score was calculated as follows: score 0–5, lowest adherence; score 6–9, average adherence and score ≥10, highest adherence [26], as already reported [27,28,29,30,31].

### 2.4. Anthropometric Measurements

Subjects dressed in light clothes and no shoes when anthropometric parameters were assessed. The formula of the BMI was the following: weight (kg)/height (m^2^). A wall-mounted stadiometer was used to assess height. A calibrated scale was used to assess body weight. Waist circumference (WC) was measured to the closest 0.1 cm with a no extensible tape. In all individuals, a sphygmomanometer (Gelman Hawksley Ltd., Sussex, UK) was used to measure systolic (SBP) and diastolic (DBP) blood pressure after the subject rested for a minimum of 10 min.

### 2.5. Laboratory Test

After fasting overnight, the venous blood samples were collected from the antecubital vein and was stored in vacutainer tubes containing ethylenediaminetetraacetic acid (EDTA). A Roche Modular Analytics System was used to assess all biochemical parameters—including total cholesterol and triglycerides (TG), Alanine Transaminase (ALT), Aspartate Aminotransferase (AST), and γ-Glutamyltransferase (γGT). Low-density lipoprotein (LDL) and high-density lipoprotein (HDL) cholesterol were assessed by a direct method (homogeneous enzymatic assay for the direct quantitative determination of LDL and HDL cholesterol). The glucose oxidase method was used to assess fasting plasma glucose. Fasting insulin levels were measured by a solid-phase chemiluminescent enzyme immunoassay using commercially available kits (Immunolite Diagnostic Products Co., Los Angeles, CA, USA). The intra-assay coefficients of variations (CV) was <5.5%. A c-reactive protein (CRP) levels were determined with a nephelometric assay with CardioPhase high-sensitive from Siemens Healthcare Diagnostics (Marburg, Germany). The intra-assay CV for CRP was <4%; low detection limit was >0.1 mg/L. The GH/IGF-1 axis was evaluated by measuring GH peak after GHRH + ARGININE (ARG) and assay of circulating IGF-1 levels. The GHRH (1–29, Geref, Serono, Rome, Italy) + ARG (arginine hydrochloride, Salf, Bergamo, Italy) test was performed according to Ghigo et al. [32]. The GH response after GHRH + ARG was classified as deficient when GH peak was ≤4.2 mg/L and sufficient when GH peak was ≥4.2 mg/L [33]. Serum GH levels were measured by immunoradiometric assay (IRMA) using commercially available kits (HGH-CTK-IRMA, Sorin, Saluggia, Italy). The sensitivity of the assay was 0.02 mg/L. The intra- and inter-assay coefficients of variations (CVs.) were 4.5% and 7.9%, respectively. IGF-1 levels were classified as deficient when the standard deviation scores (SDS) from the mean was <−2 for age and gender and sufficient when the SDS ranged from >−2 to 2.24 [34]. Serum IGF-1 levels were measured by IRMA after ethanol extraction (DSL Inc., Webster, TX, USA); assay sensitivity was 0.8 mg/L. The intra-assay CVs were 3.4%, 3.0% and 1.5% for the low, medium and high points of the standard curve, respectively. The interassay CVs. were 8.2%, 1.5% and 3.7% for the low, medium and high points of the standard curve, respectively. Homeostasis Model Assessment of Insulin Resistance (HoMA-IR) was calculated as previously reported [12].

### 2.6. Bioelectrical Impedance Analysis

Bioelectrical Impedance Analysis (BIA) body composition was assessed using a BIA phase-sensitive system by experienced observers (an 800-µA current at a signal-frequency of 50 kHz BIA 101 RJL, Akern Bioresearch, Florence, Italy) [35], as previously reported [30,31,36,37]. The exam was performed as suggested by the European Society of Parental and Enteral Nutrition (ESPEN) [38]. Electrodes were placed on the hand and the ipsilateral foot, according to Kushner [39]. The phase angle (PhA) was obtained from conditions under 50 kHz according to the following formula: PhA (°, degrees) = arctangent reactance (Xc)/resistance (R) × (180/π).

### 2.7. Visceral Adiposity Index

The visceral adiposity index (VAI) has been calculated by the following formula, with triglycerides levels expressed in mmol/L and HDL levels expressed in mmol/L [40]:

VAI (Female): [WC/36.58 + (1.89 × BMI)] × (TG/0.81) × (1.52/HDL).

### 2.8. Fatty Liver Index

The fatty liver index was calculated according to the following formula [41]:

[100 × exp [0.953 × ln(TG) + 0.139 × BMI + 0.718 × ln(γ-glutamiltransferase) + 0.053 × [WC] − 15.745]/(1 + exp[0.953 × ln(TG) + 0.139 × BMI + 0.718 × ln(γ-glutamiltransferase) + 0.053 × [WC] − 15.745].

### 2.9. Criteria to Define Metabolic Syndrome

According to the National Cholesterol Education Program (NCEP) and Adult Treatment Panel (ATP III) definition, a metabolic syndrome is present if three or more of the following five criteria are met: WC over 88 cm in female, respectively), blood pressure over 130/85 mmHg, fasting TG level over 150 mg/dL, fasting HDL cholesterol level less than 50 mg/dL (in female) and fasting glucose over 100 mg/dL (NCEP-ATP III 2000) [41].

### 2.10. Statistical Methods

The variable distribution was assessed by Kolmogorov-Smirnov test and results were reported as mean ± SD or as median plus range. Mann–Whitney U test, or Student’s unpaired *t*-test, was used to assess differences between groups. The significance of differences in frequency distributions was assessed by chi2 (χ^2^) test. Pearson r or Spearman’s rho correlation coefficients were used to assess correlations between variables. In order to investigate the association among quantitative variables (all food items of the PREDIMED questionnaire and PREDIMED score), bivariate proportional odds ratio (OR) models, 95% interval confidence (IC), and adjusted R2 were performed. In these analyses, we only entered those variables that had a *p* value of <0.05 in the univariate analysis (partial correlation). To avoid multicollinearity, variables with a variance inflation factor (VIP) >10 were excluded. Values ≤5% were considered statistically significant. Data were stored and analyzed using the MedCalc^®^ package (Version 12.3.0 1993–2012 MedCalc Software bvba-MedCalc Software, Mariakerke, Belgium). The sample size calculations were performed using G POWER software. Cohen’s d (effect size) was determined by calculating the mean difference of IGF-1 levels between obese individuals with and without GH deficiency (GHD) and then dividing the result by the pooled standard deviation [12]. The resulting total minimum sample size—estimated according to a global effect size of 0.7 with type I error of 0.05 and a power of 95%—was 118 subjects. We enrolled 200 subjects to prevent any drop-outs.

## 3. Results

We report sociodemographic, anthropometric and metabolic characteristics of 200 female subjects with obesity in Table 1. Obesity was present in most of the enrolled subjects. In particular, moderately obesity was detected in 41 subjects (20.5%) while severe obesity was detected in 159 individuals (79.5%). Based on GH peak response and IGF-1 SDS, an altered GH peak and IGF-1 deficiency was found in 123 individuals (61.5%) and 142 individuals (71%), respectively. Insulin-resistance was found in 77.5% (155 subjects), while 44% (88 subjects) had metabolic syndrome. Sociodemographic, anthropometric measurements and metabolic characteristics of 200 obese female participants according to GH status are reported in Table 2. We compared the enrolled subjects according to GH peak response. The same subjects were compared according to IGF-1 SDS as well. As expected, those who were found to have both a blunted GH peak response and IGF-1 deficiency showed worst anthropometric measurements and metabolic profile than obese counterparts with normal GH peak response or with IGF-1 sufficiency.

Response frequency of dietary components included in the PREDIMED questionnaire of the subjects at baseline was reported in Table 3. We found that patients with blunted GH peak response consumed less frequently extra virgin olive, fruits, legumes and fish/seafood compared to subjects with normal GH peak response. When the same patients were divided according to IGF-1 SDS, we found that patients with IGF-1 deficiency consumed less fruits and fish/sea food and more soda drink and commercial sweets and confectionery compared to subjects with IGF-1 sufficiency. According to the PREDIMED score a higher percentage of subjects with low adherence to the Mediterranean Diet was found in subjects with blunted GH peak response and when divided according to IGF-1 SDS, low adherence was found in patients with IGF-1 deficiency, compared to their normal counterparts.

In Table 4 we reported the total energy and the daily macronutrients intake obtained from the seven-day food records. Although there was no difference in terms of total energy intake, both blunted GH peak response and IGF-1 deficiency were associated with a lower intake of proteins and a higher intake of carbohydrates, compared to their normal counterparts. Further, IGF-1 deficiency was associated with a higher intake of fats compared to subjects with IGF-1 sufficiency. Body composition parameters evaluated by the BIA are shown in Table 4 as well. In particular, both blunted GH peak response and IGF-1 deficiency were associated with lower values of phase angle (PhA) (*p* < 0.001), intra-cellular water (ICW) (%), and a higher values of extra-cellular water (ECW) compared to their normal counterparts. Additionally, a lower fat free mass (both absolute and percentage) along with a higher fat mass (both absolute and percentage) were detected.

### Correlation Studies

Correlation analysis were performed to assess the association of GH peak and IGF-1 levels with anthropometric measures and metabolic profile. As expected, both GH peak and IGF-1 levels were inversely associated with anthropometric measurements, SBP/DBP, fasting glucose, fasting insulin, HoMA-IR, total cholesterol, LDL cholesterol, fasting TG, γ-glutamiltransferase (γGT), C reactive protein, VAI, FLI and the percentage of metabolic syndrome. A direct association was found between HDL cholesterol and both GH peak and IGF-1 levels (Table 5). Both GH peak response and IGF-1 levels positively correlated to protein intake while they negatively correlated to carbohydrate and fat intake (Table 6). The correlation analysis between body composition and both GH peak response and IGF-1 levels are showed in Table 6.

In Table 7 the results of bivariate proportional odds ratio model performed to assess the association of GH peak and IGF-1 levels with food items of PREDIMED questionnaire were summarized. On the contrary, the highest odds of soda drinks and commercial sweets and confectionery appeared to have a negative effect on both GH peak and IGF-1 levels. A positive association was found between the consumption of nuts and GH peak response; a positive correlation was found between the consumption of poultry and IGF-1 levels.

**Figure 1:**
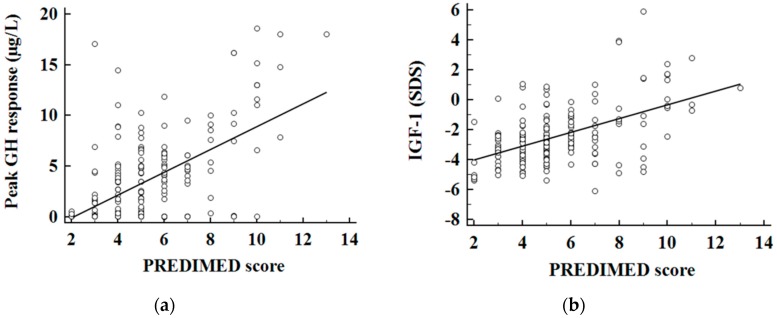
Correlation between PREDIMED (PREvention with MEDiterranean Diet) score and GH peak (**a**) and IGF-1 SDS (**b**). Growth hormone (GH); insulin-like growth factor I (IGF-1); standards deviations scores (SDS).

**Figure 2:**
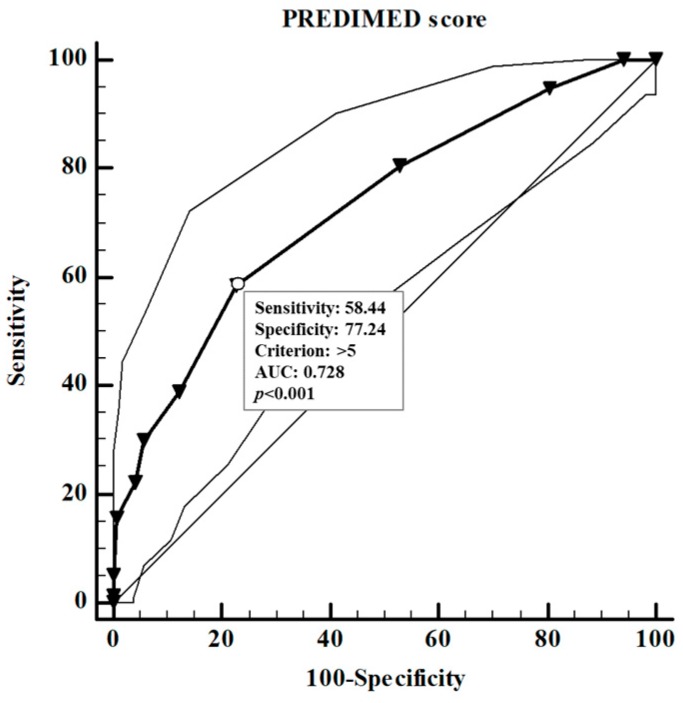
Identification of a cut-off value of PREDIMED score predicting a blunted GH peak response. A value of PREDIMED score of ≤5.0 (*p* < 0.001, AUC 0.728) could serve as a threshold for a significantly decrease of GH peak response. A p value in bold type denotes a significant difference (*p* < 0.05). Growth hormone (GH); area under the curve (AUC).

In addition, a positive association was found between the adherence to the Mediterranean Diet and both GH peak response (Figure 1a) and IGF-1 levels (Figure 1b). To evaluate the predictive value of: (a) nutritional parameters; (b) cardiometabolic indices; (c) metabolic syndrome and (d) BIA parameters on GH peak response (µg/L), we performed two multiple linear regression analysis models, which included PREDIMED score and proteins intake (model I) and measures of the body composition parameters (model II). Using model I, both PREDIMED score and proteins intake entered at the first step (*p* < 0.001) appeared to exert a powerful influence on GH peak response, while the other variables (EVOO, vegetables, fruits, soda drinks, legumes, fish/seafood, commercial sweets and confectionery, tree nuts, carbohydrates and fats) were excluded. Using model II, FLI, FM and PhA were entered at the first step (*p* < 0.001) and appeared to be the most powerful factors influencing GH peak response while the other variables (HoMA-IR, VAI, metabolic syndrome, and other parameters of the BIA) were excluded. The results of the two models are shown in Table 8. ROC analysis was performed to determine the cut off values of the adherence to the Mediterranean Diet that were predictive of highest GH peak response (Figure 2). A value of PREDIMED score of ≤5.0 (*p* < 0.001, AUC 0.728) could serve as a threshold for a significant decrease of GH peak response.

## 4. Discussion

The novel findings of this cross-sectional observational study are that both the blunted GH peak response and/or IGF-1 deficiency are associated with a worst body composition and cardiometabolic profile in obesity. Further, nutritional status, in particular the degree of adherence to the Mediterranean Diet and protein intake has been found to be associated with GH peak response in obese patients. Based on ROC curve analysis, a PREDIMED score of ≤5.0 was associated with derangements of GH peak response. Both blunted GH response and IGF-1 deficiency were damage indicators of somatotropic axis and resulted in similar cardiometabolic derangements. In fact, in this study, both blunted GH response and IGF-1 deficiency were associated with an increased fat mass and a decreased free fat mass. These findings were in agreement with previous studies performed in subjects with GHD in which changes in body composition were characterized by reduced lean body mass and increased visceral adiposity [42,43]—a clinical phenotype which has been tightly associated with insulin resistance and glucose derangements. In view of the existing association between visceral fat mass, insulin resistance and non-alcoholic fatty liver disease (NAFLD), we found that derangements of somatotropic axis were associated with increased visceral fat and fat liver in obesity, as demonstrated by higher VAI and FLI. These findings were in line with cross-sectional data reporting an increased visceral fat mass along with increased liver fat content in patients with GHD [44,45,46]. Moreover, several case reports of hypopituitaric patients reported a reduction in liver fat content following GH replacement therapy [47,48,49]. Given the tight association between NAFLD and insulin resistance measured by HoMA-IR [50], we found that derangements of somatotropic axis were associated with increased insulin resistance. As is well known, insulin resistance is an important feature of metabolic syndrome and is associated with low-grade chronic inflammation, endothelial dysfunction and increased cardiovascular mortality [51]. So far, the prevalence of metabolic syndrome is high in GHD patients. Van der Klaauw et al. [52] reported that GHD patients had a more than a two-fold higher prevalence of metabolic syndrome compared to the general population. Attanasio et al. [53] found as well that metabolic syndrome prevalence was increased in GHD patients. In agreement with this, we found that people with obesity with derangements of somatotropic axis had an increased percentage of metabolic syndrome and low-grade chronic inflammation. Moreover, PhA, a parameter obtained from BIA direct measures, such as R and Xc, is widely used as a marker of cellular health [54]. In fact, PhA is currently considered as a predictive marker of mortality and morbidity in several diseases [55]. In a healthy population, PhA could be determined by several factors such as diet, sex, age, BMI, and inflammatory status [56]. PhA provides information on the integrity [54] of a large number of cell membranes and the water distribution in body fluids [55]. Thus, PhA is positively associated with the body cell mass [56,57,58,59] and negatively associated with ECW/ICW ratio [60]. In agreement with other studies in different chronic inflammatory diseases [22,36], in this study we evidenced that a small PhA was detected in obese patients with derangements of somatotropic axis, and we hypothesized that this could be tightly related to the inflammatory status. Of interest, among all BIA measurements, PhA along with FLI and fat mass were predictive factors of GH peak response. Nevertheless, it has been reported that dyslipidemia is one of the most important contributor to the increase of hypopituitarism-related cardiovascular risk [61,62]. Derangements of serum lipid concentrations improved after GH replacement therapy [63]. People with obesity with derangements of somatotropic axis showed a worse lipid profile characterized by increased triglycerides, total and LDL cholesterol along with a reduction of HDL cholesterol. It has been reported as well that hypertension is a common finding in GHD subjects. In line with these data we found that derangements of somatotropic axis were associated with a higher SBP and DBP compared to their healthy counterparts. In addition, we found that derangements of somatotropic axis were associated with a lower adherence to the Mediterranean Diet. In addition, by carefully evaluating their dietary assessment, people with obesity with derangements of somatotropic axis consumed higher amounts of simple carbohydrates, total fats and a lower amount of proteins. The PREDIMED score, and in particular proteins intake, were found to predict GH peak response. In particular, a value of PREDIMED score of ≤5.0 has been identified as the cut-off value associated with a significant decrease of GH peak response. It has already been reported that protein intake could be a powerful stimulus for somatotropic axis [12]. However, our results lead us to hypothesize that the cluster of food components enclosed in the Mediterranean Diet could have an addictive effect to the stimulus of protein intake. Thus, the strong point of our study is that we highlighted the therapeutic role of nutrition on the obesity-related GH/IGF-1 axis derangements. This is of paramount importance because it allows for a tailored nutritional approach in people with obesity developing this hormonal defect. The main limit of the study was the lack of control group of lean subjects. However, in order to minimize this limit, we consider people with obesity without GH/IGF-1 derangement as control.

## 5. Conclusions

In conclusion, we reported an association between the derangements of somatotropic axis and some cardiovascular risk factors in people with obesity. The nutritional status—in particular the degree of adherence to the Mediterranean Diet and proteins intake—was found to be one of the most predictive factor of GH status in obesity, showing that a novel association exists. Specific cut-off values for the degree of adherence to the Mediterranean Diet could be included as an auxiliary tool in the complex obesity evaluation, contributing to identify those patients who could get additional benefit from careful evaluation of somatotropic axis. Further studies on a large population with obesity and derangements of somatotropic axis and intervention trials are warranted to support the association between the adherence to the Mediterranean Diet and the clinical severity of alterations of somatotropic axis.

## Figures and Tables

**Table 1:** Socio-demographic, anthropometric measures and metabolic profile of 200 adult females with obesity included in the study.

Parameters	Mean ± SD
Age (years)	36.98 ± 11.10
BMI (kg/m^2^)	45.19 ± 6.30
Grade II obesity	41, 20.5%
Grade III obesity	159, 79.5%
Waist circumference (cm)	126.74 ± 17.05
SBP (mmHg)	126.89±15.29
DBP (mmHg)	80.68 ± 10.84
GH peak response (µg/L)	3.72 ± 4.29
GH deficit ^1^	123, 61.5%
IGF-1 (µg/L)	151.93 ± 64.93
IGF-1 (SDS)	−2.43 ± 1.85
IGF-1(SDS) deficiency ^2^	142, 71.0%
Fasting glucose (mg/dL)	105.27 ± 36.79
Fasting insulin (µU/mL)	19.95 ± 11.76
Total cholesterol (mg/dL)	187.36 ± 47.88
HDL cholesterol (mg/dL)	50.17 ± 11.94
LDL cholesterol (mg/dL)	106.54 ± 49.96
Fasting triglycerides (mg/mL)	151.95 ± 53.10
AST (U/L)	30.15 ± 16.44
ALT (U/L)	41.70 ± 30.21
γGT (U/L)	41.37 ± 25.38
CRP (ng/mL)	2.33 ± 1.01
HoMA-IR	6.04 ± 6.55
>Cut-off	155, 77.5%
VAI	1.86 ± 0.94
>Cut-off	57, 28.5%
FLI	96.43 ± 6.79
>Cut-off	197, 98.5%
Metabolic syndrome (presence)	88, 44.0%

^1^ GH (growth hormone) peak response after GHRH (GH releasing hormone) + ARG (arginine hydrochloride) was classified as deficient (GH deficit ^1^) when GH peak was ≤4.2 mg/L. ^2^ IGF-1 levels were classified as deficient (IGF-1 (insulin-like growth factor I) deficiency ^2^) when the SDS from the mean was <−2 for age and gender.

**Table 2:** Anthropometric measures and metabolic profile of 200 adult females with obesity according to blunted GH peak or IGF-I SDS.

Parameters	Blunted GH Peak ^1^ *n* = 123, 61.5%	Normal GH Peak ^2^ *n* = 77, 35.8%	*p*-Value	IGF-1 Deficiency ^3^ *n* = 142 (71.0%)	IGF-1 Sufficiency ^4^ *n* = 58 (29.0%)	*p*-Value
BMI (kg/m^2^)	47.47 ± 6.05	41.54 ± 4.84	<0.001	46.61 ± 6.06	41.70 ± 5.53	<0.001
Waist circumference (cm)	132.11 ± 16.33	118.18 ± 14.60	<0.001	129.98 ± 16.09	118.83 ± 16.88	<0.001
SBP (mmHg)	130.83 ± 15.41	120.58 ± 12.87	<0.001	129.19 ± 15.17	121.24 ± 14.21	0.001
DBP (mmHg)	83.33 ± 9.32	76.45 ± 11.77	<0.001	82.16 ± 10.02	77.05 ± 11.94	0.002
GH peak response (µg/L)	-	-	-	2.14 ± 2.78	7.59 ± 4.88	<0.001
IGF-1 (µg/L)	120.94 ± 44.41	201.45 ± 61.91	<0.001	-	-	-
IGF-1 (SDS)	−3.26 ± 1.12	−1.11 ± 2.04	<0.001	-	-	-
Fasting Glucose (mg/dL)	115.00 ± 40.41	89.73 ± 22.94	<0.001	111.67 ± 39.12	89.66 ± 24.32	<0.001
Fasting Insulin (µU/mL)	24.47 ± 11.59	12.73 ± 7.77	<0.001	22.50 ± 11.52	13.71 ± 9.93	<0.001
Total cholesterol (mg/dL)	195.06 ± 48.20	175.08 ± 45.00	0.004	192.02 ± 47.82	175.97 ± 46.49	0.031
HDL cholesterol (mg/dL)	48.09 ± 11.41	53.47 ± 12.10	0.002	48.59 ± 11.57	54.00 ± 12.07	0.004
LDL cholesterol (mg/dL)	114.55 ± 50.28	93.77 ± 47.00	0.004	111.39 ± 49.88	94.68 ± 48.55	0.031
Fasting Triglycerides (mg/dL)	159.94 ± 53.09	139.19 ± 50.89	0.007	158.30 ± 52.05	136.37 ± 52.87	0.008
AST (U/L)	31.17 ± 16.64	28.52 ± 16.08	0.268	30.25 ± 16.36	29.91 ± 16.77	0.897
ALT (U/L)	41.54 ± 32.53	41.96 ± 26.27	0.919	41.56 ± 31.25	42.03 ± 27.75	0.921
γGT (U/L)	42.26 ± 22.50	37.14 ± 19.31	0.089	42.34 ± 21.61	35.28 ± 20.27	0.030
CRP (ng/mL)	2.46 ± 1.02	2.12 ± 0.96	0.019	2.47 ± 1.00	1.98 ± 0.95	0.002
HoMA-IR	7.81 ± 7.07	3.20 ± 4.36	<0.001	7.08 ± 6.83	3.49 ± 5.02	<0.001
VAI	2.06 ± 1.01	1.55 ± 0.69	<0.001	2.00 ± 0.96	1.50 ± 0.75	<0.001
FLI	98.23 ± 3.04	93.45 ± 9.61	<0.001	97.98 ± 3.18	92.64 ± 10.75	<0.001
Presence of Metabolic Syndrome (*n*, %)	75, 61.0%	13, 16.9%	<0.001	75, 52.8%	13, 22.4%	<0.001

^1^ GH peak response after GHRH + ARG was classified as blunted when GH peak was ≤4.2 mg/L, and ^2^ sufficient when GH peak was ≥4.2 mg/L. ^3^ IGF-1 levels were classified as deficient (IGF-1 deficiency) when the SDS from the mean was <−2 for age and gender, and ^4^ sufficient when the SDS ranged from >−2 to 2.24.

**Table 3:** Response frequency of dietary components included in the PREDIMED (PREvention with MEDiterranean Diet) questionnaire of 200 adult females with obesity included in the study divided according to blunted GH peak or IGF-1 SDS.

Questions PREDIMED Questionnaire	Blunted GH Peak ^1^ *n* = 123, (61.5%)	Normal GH Peak ^2^ *n* = 77, (35.8%)	χ^2^, *p*-Value	IGF-1 Deficiency ^3^ *n* = 142, (71.0%)	IGF-1 Sufficiency ^4^ *n* = 58, (29.0%)	χ^2^, *p*-Value
EVOO as main culinary lipid	57, 46.3%	54, 70.1%	9.71, 0.002	75, 52.8%	36, 62.1%	1.08, 0.299
EVOO >4 tablespoons	27, 22.0%	22, 28.6%	0.79, 0.373	32, 22.5%	17, 29.3%	0.069, 0.407
Vegetables ≥2 servings/day	34, 27.6%	32, 41.6%	3.54, 0.059	41, 28.9%	25, 43.1%	3.16, 0.076
Fruits ≥3 servings/day	32, 26.0%	48, 62.3%	24.53, <0.001	46, 32.4%	34, 58.6%	10.73, 0.011
Red/processed meats <1/day	54, 43.9%	43, 55.8%	2.25, 0.134	65, 45.8%	32, 55.2%	1.10, 0.293
Butter, cream, margarine <1/day	52, 42.3%	26, 33.8%	1.11, 0.293	56, 39.4%	22, 37.9%	0.01, 0.969
Soda drinks <1/day	39, 31.7%	30, 39.0%	0.81, 0.369	40, 28.2%	29, 50.0%	7.75, 0.001
Wine glasses ≥7/week	33, 26.8%	21, 27.3%	0.01, 0.925	37, 26.1%	17, 29.3%	0.09, 0.768
Legumes ≥3/week	56, 45.5%	47, 61.0%	3.96, 0.046	70, 49.3%	33, 56.9%	0.67, 0.412
Fish/seafood ≥3/week	20, 16.3%	45, 58.4%	36.51, <0.001	9, 6.3%	26, 96.6%	148.69, <0.001
Commercial sweets and confectionery ≤2/week	50, 40.7%	39, 50.6%	1.54, 0.216	55, 38.7%	34, 58.6%	5.82, 0.016
Tree nuts ≥3/week	16, 13.0%	15, 19.5%	1.06, 0.303	20, 14.1%	11, 19.0%	0.42, 0.516
Poultry more than red meats	51, 41.5%	39, 50.6%	1.27, 0.261	63, 44.4%	27, 46.6%	0.02, 0.900
Use of sofrito sauce ≥2/week	61, 49.6%	38, 49.4%	0.01, 0.911	69, 48.6%	30, 51.7%	0.06, 0.806
PREDIMED categories						
Low adherence to the Mediterranean Diet	95, 77.2%	32, 41.6%	24.49, <0.001	107, 75.4	20, 34.5%	27.94, <0.001
Average adherence to the Mediterranean Diet	27, 22.0%	33, 42.9%	8.89, 0.001	34, 23.9	26, 44.8%	7.59, 0.006
High adherence to the Mediterranean Diet	1, 0.8%	12, 15.6%	14.66, 0.001	1, 0.7	12, 20.7%	23.88, <0.001

^1^ GH peak response after GHRH + ARG was classified as blunted when GH peak was ≤4.2 mg/L, and ^2^ sufficient when GH peak was ≥4.2 mg/L. ^3^ IGF-1 levels were classified as deficient (IGF-1 deficiency) when the SDS from the mean was <−2 for age and gender, and ^4^ sufficient when the SDS ranged from >−2 to 2.24.

**Table 4:** Total energy and macronutrient intake of 200 adult females with obesity included in the study divided according to blunted GH peak or IGF- 1 SDS.

Parameters	Blunted GH Peak ^1^ *n* = 123, 61.5%	Normal GH Peak ^2^ *n* = 77, 35.8%	*p*-Value	IGF-1 Deficiency ^3^ *n* = 142 (71.0%)	IGF-1 Sufficiency ^4^ *n* = 58 (29.0%)	*p*-Value
**Dietary assessment**						
Total energy (kcal)	2838.97 ± 253.89	2783.68 ± 255.47	0.136	2830.21 ± 250.61	2787.00 ± 266.11	0.278
Protein (gr of total kcal)	112.92 ± 14.36	127.90 ± 17.98	<0.001	112.32 ± 13.33	134.27 ± 16.47	<0.001
Carbohydrate (gr of total kcal)	388.99 ± 38.27	376.01 ± 36.34	0.018	388.58 ± 37.19	372.78 ± 37.87	0.007
Fat (gr of total kcal)	92.36 ± 10.46	85.34 ± 9.84	<0.001	91.84 ± 10.72	84.31 ± 8.89	<0.001
**BIA parameters**						
R (Ω)	489.47 ± 95.91	471.45 ± 80.07	0.153	488.16 ± 93.22	471.56 ± 79.74	0.296
Xc (Ω)	42.20 ± 8.49	43.82 ± 7.89	0.180	42.15 ± 8.13	44.73 ± 8.82	0.082
PhA (°)	4.95 ± 0.49	5.35 ± 0.70	<0.001	4.97 ± 0.50	5.45 ± 0.79	<0.001
FM (kg)	68.05 ± 16.24	49.98 ± 12.02	<0.001	68.37 ± 16.61	45.83 ± 10.15	<0.001
FM (%)	53.30 ± 6.71	46.73 ± 6.70	<0.001	53.38 ± 6.70	45.21 ± 6.49	<0.001
FFM (kg)	58.29 ± 8.03	55.99 ± 7.37	0.041	58.41 ± 7.87	54.92 ± 7.73	0.014
FFM (%)	46.69 ± 6.71	53.28 ± 6.73	<0.001	46.62 ± 6.71	54.81 ± 6.54	<0.001
Skeletal muscle mass (kg)	29.77 ± 7.12	32.52 ± 7.54	0.011	29.88 ± 7.20	33.01 ± 8.08	0.020
Skeletal muscle mass (%)	23.96 ± 6.38	31.34 ± 8.97	<0.001	24.01 ± 6.50	33.37 ± 9.68	<0.001
TBW (Lt)	42.72 ± 5.85	40.99 ± 5.39	0.035	42.75 ± 5.76	40.19 ± 5.66	0.014
TBW (%)	34.18 ± 4.90	39.00 ± 4.93	<0.001	34.13 ± 4.90	40.12 ± 4.79	<0.001
ICW (Lt)	20.87 ± 3.35	20.87 ± 3.48	0.980	20.91 ± 3.32	20.67 ± 3.71	0.708
ICW (%)	48.77 ± 2.81	50.83 ± 3.65	<0.001	48.83 ± 2.87	51.33 ± 4.15	<0.001
ECW (Lt)	21.86 ± 2.95	20.11 ± 2.69	<0.001	21.83 ± 2.93	19.52 ± 2.88	0.001
ECW (%)	51.23 ± 2.81	49.17 ± 3.65	<0.001	51.17 ± 2.87	48.67 ± 4.15	0.001
BCMI	8.34 ± 2.36	9.69 ± 2.68	<0.001	8.37 ± 2.39	10.00 ± 2.86	0.001

^1^ GH peak response after GHRH + ARG was classified as blunted when GH peak was ≤4.2 mg/L, and ^2^ sufficient when GH peak was ≥4.2 mg/L. ^3^ IGF-1 levels were classified as deficient (IGF-1 deficiency) when the SDS from the mean was <−2 for age and gender, and ^4^ sufficient when the SDS ranged from >−2 to 2.24. R (Ω): resistance; Xc (Ω): reactance; PhA: phase angle; FM: fat mass; FFM: fat free mass; TBW: total body water; ICW: intracellular body water; ECW: extracellular body water; BCMI: body cell mass index; BIA: Bioelectrical Impedance Analysis.

**Table 5:** Correlation between GH peak response and IGF-1 SDS with Socio-demographic, anthropometric measures and metabolic profile.

	GH peak Response (µg/L)		IGF-1 (SDS)	
Parameters	r	*p* Value	r	*p* Value
Age (years)	0.158	0.026	0.315	<0.001
BMI (kg/m^2^)	−0.548	<0.001	−0.369	<0.001
Waist circumference (cm)	−0.484	<0.001	−0.331	<0.001
SBP (mmHg)	−0.354	<0.001	−0.261	<0.001
DBP (mmHg)	−0.315	<0.001	−0.303	<0.001
Fasting glucose (mg/dL)	−0.381	<0.001	−0.284	0.001
Fasting insulin (µU/mL)	−0.521	<0.001	−0.338	<0.001
Total cholesterol (mg/dL)	−0.239	0.001	−0.228	0.001
HDL cholesterol (mg/dL)	0.292	<0.001	0.273	<0.001
LDL cholesterol (mg/dL)	−0.265	<0.001	−0.246	<0.001
Fasting triglycerides (mg/dL)	−0.138	0.050	−0.143	0.043
AST (U/L)	0.026	0.714	0.055	0.443
ALT (U/L)	−0.050	0.479	0.026	0.711
γGT (U/L)	−0.134	0.050	−0.191	0.007
CRP (ng/mL)	−0.163	0.021	−0.206	0.003
HoMA−IR	−0.378	<0.001	−0.253	<0.001
VAI	−0.236	0.001	−0.217	0.002
FLI	−0.592	<0.001	−0.495	<0.001
Metabolic syndrome (*n*, %)	−0.332	<0.001	−0.255	<0.001

**Table 6:** Correlation between GH peak response and IGF-1 SDS with nutrient intake and BIA parameters.

	GH peak Response (µg/L)		IGF-1 (SDS)	
Parameters	r	*p*-Value	R	*p*-Value
**Dietary assessment**				
Total energy (kcal)	−0.100	0.158	−0.017	0.806
Protein (gr of total kcal)	0.536	<0.001	0.761	<0.001
Carbohydrate (gr of total kcal)	−0.237	0.001	−0.169	0.017
Fat (gr of total kcal)	−0.277	<0.001	−0.329	<0.001
**BIA parameters**				
R (Ω)	−0.109	0.123	−0.023	0.750
Xc (Ω)	0.120	0.090	0.155	0.029
PhA (°)	0.392	<0.001	0.280	<0.001
FM (kg)	−0.585	<0.001	−0.409	<0.001
FM (%)	−0.528	<0.001	−0.328	<0.001
FFM (kg)	−0.155	0.029	0.203	0.004
FFM (%)	0.529	<0.001	0.329	<0.001
Skeletal muscle mass (kg)	0.251	<0.001	0.111	0.120
Skeletal muscle mass (%)	0.565	<0.001	0.368	<0.001
TBW (Lt)	−0.161	0.024	−0.200	0.005
TBW (%)	0.529	<0.001	0.330	<0.001
ICW (Lt)	0.016	0.818	−0.063	0.378
ICW (%)	0.368	<0.001	0.265	<0.001
ECW (Lt)	−0.328	<0.001	−0.313	<0.001
ECW (%)	−0.368	<0.001	−0.265	<0.001
BCMI	0.333	<0.001	0.202	0.004

**Table 7:** Bivariate proportional odds ratio models performed to assess the association of GH peak response and IGF-1 SDS with the dietary components included in the PREDIMED questionnaire.

	GH peak Response (µg/L) *n* = 200				IGF-1 (SDS) *n* = 200			
Parameters	OR	*p* Value	95% IC	R^2^ adj	OR	*p* Value	95% IC	R^2^ adj
EVOO as main culinary lipid	1.12	0.003	1.04–1.21	0.05	1.19	0.037	1.01–1.40	0.02
EVOO oil >4 tablespoons	1.08	0.030	1.00–1.16	0.02	1.14	0.001	0.096–1.35	0.01
Vegetables ≥2 servings/day	1.07	0.035	1.00–1.15	0.02	1.31	0.002	1.10–1.54	0.05
Fruits ≥ 3servings/day	1.22	<0.001	1.12–133	0.13	1.36	<0.001	1.15–1.62	0.07
Red/processed meats <1/day	1.06	0.062	0.99–1.14	0.02	1.07	0.394	0.09–1.24	0.01
Butter, cream, margarine <1/day	1.01	0.753	0.95–1.07	0.01	1.07	0.420	0.91–1.24	0.01
Soda drinks <1/day	1.09	0.014	1.02–1.16	0.03	1.19	0.035	1.01–1.38	0.02
Wine glasses ≥7/week	0.98	0.946	0.93–1.07	0.01	0.99	0.863	0.83–1.16	0.01
Legumes ≥3/week	1.07	0.049	1.00–1.15	0.02	1.16	0.066	0.99–1.35	0.02
Fish/seafood ≥3/week	1.36	<0.001	1.23–1.51	0.24	6.98	<0.001	3.83–12.73	0.48
Commercial sweets and confectionery ≤2/week	1.11	0.002	1.04–1.19	0.05	1.22	0.013	1.04–1.44	0.03
Tree nuts ≥3/week	1.08	0.049	1.00–1.17	0.02	0.17	0.092	0.97–1.41	0.01
Poultry more than red meats	1.05	0.089	0.99–1.13	0.01	1.17	0.042	1.00–1.37	0.02
Use of sofrito sauce ≥2/week	1.01	0.716	0.095–1.08	0.01	0.09	0.087	0.085–1.15	0.01
**PREDIMED categories**								
Low adherence to the Mediterranean Diet	0.81	<0.001	0.75–0.88	0.15	0.61	<0.001	0.50–0.75	0.14
Average adherence to the Mediterranean Diet	1.06	0.046	0.99–1.14	0.02	1.23	0.014	1.04–1.44	0.03
High adherence to the Mediterranean Diet	1.47	<0.001	1.26–1.69	0.19	1.96	<0.001	1.45–2.63	0.12

**Table 8:** Multiple regression analysis models (stepwise method) with GH Peak response (µg/L) as dependent variable to estimate the predictive value of: nutritional parameters (model 1); fatty liver index and BIA parameters (model 2).

Parameters	Multiple Regression Analysis			
**Model 1**	**R^2^**	***β***	**t**	***p*** **value**
**PREDIMED score**	0.297	0.548	9.22	<0.001
**Protein (gr of total kcal)**	0.398	0.362	5.86	<0.001
Variables excluded: EVOO, vegetables, fruits, soda drinks, legumes, fish/seafood, commercial sweets and confectionery, tree nuts, carbohydrates and fat.				
**Model 2**				
**FLI**	0.347	−0.592	−10.34	<0.001
**FM**	0.453	−0.382	−6.22	<0.001
**PhA (°)**	0.479	0.181	3.30	<0.001
Variables excluded: HoMA-IR, VAI, metabolic syndrome, and other parameters of the BIA				

Growth hormone (GH); metabolic syndrome; bioelectrical impedance analysis (BIA); phase angle (PhA); fat mass (FM); extra virgin olive oil (EVOO); homeostatic model assessment for insulin resistance (HoMA-IR); visceral adiposity index (VAI); fatty liver index (FLI).

## Abbreviations

GH	Growth hormone
IGF-I	Insulin-like growth factor I
GHD	Growth hormone deficiency
BMI	Body mass index
BIA	Bioelectrical impedance analysis
FLI	Fatty liver index
VAI	Visceral adiposity index
NAFLD	Non-alcoholic fatty liver disease
PhA	Phase angle
FM	Fat mass
FFM	Free fat mass
